# Clinicians’ Selection Criteria for Video Visits in Outpatient Care: Qualitative Study

**DOI:** 10.2196/jmir.9851

**Published:** 2018-11-05

**Authors:** Linda Sturesson, Kristina Groth

**Affiliations:** 1 Department of Learning, Informatics, Management and Ethics Karolinska Institutet Stockholm Sweden; 2 Centre for Innovation Karolinska University Hospital Stockholm Sweden; 3 Department of Clinical Science, Intervention and Technology Karolinska Institutet Stockholm Sweden

**Keywords:** outpatient care, selection criteria, telemedicine, telehealth, ethnography

## Abstract

**Background:**

Video visits with patients were introduced into outpatient care at a hospital in Sweden. New behaviors and tasks emerged due to changes in roles, work processes, and responsibilities. This study investigates the effects of the digital transformation—in this case, how video visits in outpatient care change work processes and introduce new tasks—to further improve the concept of video visits. The overarching goal was to increase the value of these visits, with a focus on the value of conducting the treatment for the patient.

**Objective:**

Through the real-time, social interactional features of preparing for and conducting video visits with patients with obesity, this study examines which patients the clinicians considered suitable for video visits and why. The aim was to identify the criteria used by clinicians when selecting patients for video visits to understand what criteria the clinicians used as the grounds for their selection.

**Methods:**

Qualitative methods were used, including 13 observations of video visits at 2 different clinics and 14 follow-up interviews with clinicians. Transcripts of interviews and field notes were thematically analyzed, discussed, and synthesized into themes.

**Results:**

From the interviews, 20 different arguments for selecting a specific patient for video visits were identified. Analyzing interviews and field notes also revealed unexpressed arguments that played a part in the selection process. The unexpressed arguments, as well as the implicit reasons, for why a patient was given the option of video visits can be understood as the selection criteria for helping clinicians in their decision about whether to offer video visits or not. The criteria identified in the collected data were divided into 3 themes: practicalities, patient ability, and meeting content.

**Conclusions:**

Not all patients with obesity undergoing treatment programs should be offered video visits. Patients’ new responsibilities could influence the content of the meeting and the progress of the treatment program. The selection criteria developed and used by the clinicians could be a tool for finding a balance between what the patient wants and what the clinician thinks the patient can manage and achieving good results in the treatment program. The criteria could also reduce the number and severity of disturbances and limitations during the meeting and could be used to communicate the requirements they represent to the patient. Some of the criteria are based on facts, whereas others are subjective. A method for how and when to involve the patient in the selection process is recommended as it may strengthen the patient’s sense of responsibility and the relationship with the clinician.

## Introduction

Telemedicine solutions can be beneficial for different stakeholders at different levels and from different perspectives [1-3]. Through telemedicine, access to health care for all can be possible as the participants are not bound to a specific place, and care can be provided for patients who struggle to physically visit health care premises, for example, because they live far away or are not strong enough to travel. Hence, there is an expectation that digital communication will, to some extent, be more inclusive; for example, marginalized groups may gain benefits [4].

One telemedicine solution is video-mediated meetings or consultations (hereafter called video visits) between patients (including relatives) and clinicians, which introduce a new way of conducting such meetings. Video visits can be as effective as face-to-face interventions [3], and they appear to be safe and convenient in outpatient care, but there are complex challenges related to their adoption by clinicians [5]. Implementing telemedicine changes how work is organized in terms of roles, tasks, and processes [5], which in turn changes the power relationships between participants and their expectations of each other [6-9]. Video visits imply that technology is used for mediating the meeting, that there is a geographical separation between the clinician and the patient [10,11] and that a nonclinical space (the patient’s) is added to the meeting [10,12]. During a video visit, the place chosen by the patient might influence the complex communication between the clinician and patient [12,13]. Introducing a nonclinical setting may affect those involved, the consultation, and the outcome of the meeting for example, because a health care environment manifests social orders [7] and facilitates the maintenance of professional and patient roles. In addition, limitations and disturbances may occur due to the technology, the patient’s surroundings, or the procedures deployed when conducting video visits. These are all aspects that might affect the behaviors of the patient and clinician, how the video visit is conducted, and its outcome [12].

Aspects that may positively influence the outcome of video visits have been identified; for example, there is an established relationship and trust between the clinician and patient, there is a need for frequent contact, and those involved in the video visit need to have the necessary technical skills [5]. Other aspects that can influence whether or not a patient is considered suitable for telemedicine solutions are, for example, the patient’s preferences and circumstances and system capacity [13], as well as how the technology may affect the patient’s health condition [5]. It is recommended that patients living a long distance from the health care premises are offered telemedicine solutions, and physicians are recommended to begin with less complex cases [13]. The selection of patients for video visits may hence be determined by various criteria such as the complexity of the meeting, the patient’s preferences and distance from the clinic, the clinician’s experience with the technology, and so forth [2]. It is known that the physical clinical environment affects patient satisfaction, attitudes, and work performances [14], which implies that selecting patients for video visits needs careful consideration. Some patients may not be considered suitable for video visits [5,13], and this introduces the new task of choosing whether a patient is suitable when implementing video visits [5]. But how do clinicians choose patients for video visits? It has been reported that patients are more positive about *tele-homecare* (which, as well as video visits, also includes daily monitoring of data) than clinicians, and their perceptions of such care may differ from that of clinicians [15]. Even though *tele-homecare* includes daily monitoring of tasks, the perception of a video visit may also differ between patients and clinicians. The perception may also be influenced by the type of care and issues addressed during the video visit. In this study, we explored the specific task of selecting patients for video visits as part of a treatment program in outpatient care at a university hospital in Sweden. Video visits were not offered through spontaneous meetings initiated by the patients [5].

The aim of this study was to identify the criteria used by clinicians when selecting patients for video visits, by exploring which patients the clinicians considered suitable for video visits and why. The study was part of a broader investigation including how video visits in outpatient care change work processes and introduce new tasks, with the overarching goal of increasing the value of these visits by making further improvements to the concept. The value may differ between stakeholders, and our focus was on the treatment of the patient—not only on the value of benefits to the patient but also for the hospital and society. The value can be measured by the progress of the treatment.

## Methods

### Overview

The study was qualitative and exploratory in its approach. Interviews with clinicians and observations of video visits were conducted to generate data. The focus was on the situatedness in the use of video visits and the situated actions when clinicians conducted such visits [16]. In addition, both formal and informal settings in everyday work and ad hoc individual conversations related to video visits were observed and analyzed to understand the phenomenon of video visits and their role in a wider context.

Our analysis resulted in 2 overarching categories: the criteria used by clinicians when selecting patients for video visits and disturbances and limitations. The first category is presented in this paper, and the second has been addressed in another paper [12]. As the 2 categories result from the same research study, the same methods and materials were used.

### Approach to the Research Area

Theoretical perspectives in symbolic interactionism provided a source of inspiration and a starting point, providing frameworks suitable for analyzing social reality and understanding human behavior and human feelings. Social interaction can be influenced by moods, weather, locations, and environments [17]. The individual defines the situation both consciously and unconsciously, and human behavior is seen in relation to the whole context [17]. Both diversity and commonalities are sought with an open mind, with attention being given “to what falls out of view or falls between the cracks” [18].

In our study, the video visits were part of a treatment program that included several consecutive meetings. The consultation is a social interaction that involves a clinician, a patient and often a relative, in which at least 1 of them has a predetermined goal for the meeting. What happens between those involved can be understood as social acting and, more specifically, as an instrumental or planned action [16]. For example, a clinician may have the goal of learning about the patient’s behavior since the last meeting, progress, side effects, etc. To achieve this goal, the clinician will prepare by reading the patient’s medical record and making notes on what to address during the consultation. However, each consultation session is a link in a longer treatment chain—a path along which each situation affects the outcome of each session [16].

Clinicians develop skills based on physical consultations, and face-to-face visits become the norm for clinical meetings [9]. The clinicians’ frame of reference is thus the traditional physical meeting or a follow-up by phone. When introducing video visits, clinicians are therefore likely to compare them with traditional clinical meetings. In our study, we explored video visits by gathering examples of the clinicians selecting patients for such visits, the reasons behind their choices, and how they shared their experiences and discussed their choices.

### Ethical Approval and Consent 

Ethical approval for the study was given by the Regional Ethical Review Board in Stockholm before data gathering (reference number: 2016/1027-31). The clinicians obtained written informed consent for participation and for publication (including information about participation, anonymity, the purpose and objectives of the study, and the responsible researcher) from patients, relatives, and guardians. Participants were offered video visits instead of physical meetings. The consent form was either sent by email or given by hand to the patient and, if applicable, to relatives. The clinicians signed a written consent for participation following review by the researcher.

### Context

A total of 2 patient flows were involved in this study, named Clinic A and Clinic B. Both clinics treated patients with obesity. The clinics had congruent goals, agendas, and philosophies for their treatment. The content of care was mainly based on a humanistic perspective of health and disease, with a lesser focus on biomedical data such as weight and body composition. However, these variables were still used as treatment outcome assessments. Video visits at the clinics were part of a treatment program that included several consecutive meetings aimed at helping patients to implement lifestyle changes successfully. Clinicians supported patients in their efforts to achieve behavioral and lifestyle changes. Between visits, patients were asked to work actively on these changes by themselves. Both clinics shared the same view about using video visits as complements to face-to-face visits and for follow-ups. The staff consisted of doctors, nurses, psychologists, nutritionists, occupational therapists, and physiotherapists. Clinicians at Clinic B also had competence in cognitive behavioral therapy. Differences between the clinics are described in Table 1.

### Technology and Devices

The concept used for video visits was developed for less complex meetings in outpatient care. The technology included an ordinary videoconferencing tool with encrypted communication, capable of producing adequate quality for seeing and hearing each other and for sharing documents. The technology could not be used to connect sensors used for monitoring parameters, and the quality of the video was not high enough to provide details of problems such as skin issues. A complex video visit, such as when a neurologist needs to see small detailed movements during care for patients with Parkinson disease or to demonstrate exercises to a patient [19], may require equipment of higher quality as well as additional space in front of the video camera for specific exercises.

The patient or relative typically used his or her own device such as a computer, mobile phone, or tablet with a webcam, speaker, internet connection, and Web browser or the videoconferencing app.

### Respondents and Recruitment

In preparation for the study, 2 clinics were selected to participate. They were identified from the second author’s work with introducing video visits in outpatient care settings at the hospital. One clinic was selected because it had successfully adopted the concept of video visits earlier in the year, and the other was selected because it had shown interest and carried out test video visits but had not yet started. Moreover, 2 clinicians from the first clinic, who were already conducting video visits, and 6 clinicians from the second clinic who wanted to start video visits, agreed to participate in the study.

At both clinics, the staff selected patients or relatives for video visits. Video visits were only offered to patients who were physically present at the clinic at the beginning of their treatment. The clinicians offered video visits to the selected patients either during a physical meeting or through a telephone contact. The patients had the opportunity to accept or decline video visits. During the study period, there were patients who declined. The clinicians who conducted the video visits had previously met face-to-face with the patients.

If patients accepted a video visit, the clinicians asked them if they wanted to participate in the research study. The question was asked to the patient and, if applicable, to the relative during a face-to-face meeting, phone call, or previous video visit (at Clinic B, where video visits were being used before the research study started). The staff, patients, and any guardian provided written informed consent to participate in the research.

### Data Collection

The data collection, conducted by the first author, consisted of a total of 13 observations and 14 interviews; see Table 2 for more details. In all, 6 clinicians conducted 2 video visits each and were, therefore, observed and interviewed twice. However, 1 of the interviews was conducted without an observation (see below), resulting in a total of 13 observations and 14 interviews.

Each observation started before the actual video visit and included the time for the clinician’s immediate preparation. The researcher was located in the same room as the clinician and was visually and verbally presented to the patient and relative at the beginning of the video visit, giving each patient a chance to withdraw his or her consent. During the video visit, the researcher observed the meeting from a position out of sight of the webcam, that is, the patient and relative could not see the researcher. The observations were partly exploratory and partly structured. Some aspects such as start and end time, patient’s location, and number of participants were predetermined and noted in the observation protocol. These were combined with field diaries that contained the exploratory observation notes. The observations were not recorded, photographed, or filmed.

The interviews were in-depth, contextual, and semistructured and were conducted with the clinicians after, and in addition to, each video visit. Of the 14 interviews, 13 were conducted face-to-face and one through phone. Furthermore, 1 of the interviews occurred without an observation, as the patient withdrew consent to participate in the study as the observation was about to start. The interview was still conducted after the video visit. The interviews were recorded and transcribed verbatim.

In addition, the first author attended formal encounters (eg, treatment conferences with clinicians) as a passive observer and participated in informal gatherings (eg, lunches and other breaks), taking field notes to capture the clinical discourse and clinicians’ perceptions and thoughts about video visits, without interfering in the discussions taking place. All data were gathered during a contiguous period of 3 months during 2016. 

### Analysis

The analysis process follows a qualitative approach [20] in which the transcripts of interviews and field notes were read through several times to achieve a familiarization with the content. During the reading, themes were identified and noted in a blank document. Corresponding transcripts and field notes were read iteratively to gain a full picture of the collected data and then a conceptual framework was created. After this initial process, the transcripts of interviews and field notes were analyzed thematically [21]. The data were then read through again and coded to match the themes in the developed conceptual framework.

Spreadsheets were used to organize and sort the data. To find and keep track of patterns in the material, themes were separated into different rows in the spreadsheet, and each interview and the corresponding observations were sorted into different columns. Pieces of the text were sorted to the appropriate cells. The principle of spreadsheets was also used to analyze and find patterns in the quantitative data. The data, themes, and sorting were continuously discussed throughout the analysis.

The themes were synthesized into 2 overarching categories: “Selecting patients for video visits” and “Disturbances and limitations.” From the analysis, it became clear that selecting patients had added a new task for clinicians, and video meetings had introduced disturbances and limitations related to both the technology and the surroundings. This paper focuses on the first category “Selecting patients for video visits” and the criteria the clinicians used in the selection process. The themes sorted under the category “Selecting patients for video visits” were issues of the patient’s ability, practical matters, and the meeting content. Each theme represents a number of criteria.

In the Results section, quotes are used to illustrate situations in which selection considerations were made. The quotes chosen represent situations that occurred once or several times, illustrating an effect of something that may occur in other situations. When illustrating a situation related to the criteria with excerpts from the data, we use the notation Clinic X, Int_Y, or Obs_Y, where Int stands for interview and Obs for observation. The interview that followed an observation of a video visit was given the same number as the observation, that is, the number of the video visit.

**Table 1:** Comparison of patient population, implementation stages, and settings of Clinic A and Clinic B.

Aspect of the setting	Clinic A	Clinic B
Patient population	Children and adolescents with obesity (aged 2-18 years).	Adults with obesity (aged >18 years).
Responsible for and involved in the treatment	Relatives were responsible for treatment and provided an important role in its implementation. Relatives of young children visited the clinic together with the child. Follow-ups and reconciliations were made by phone with relatives of young children and not with the child. Teenage patients were assessed by the clinicians to decide whether they were mature enough to take responsibility for their own treatment. If so, the relative usually did not participate in follow-ups.	Patients were responsible for their own treatment. Relatives were not present during meetings.
Stages of implementation of video visits	Video visits began when the research study started.	6-month history of carrying out video visits.
Setting for video visits	One room was used for video visits. The room was equipped with a computer, camera, and headset. Clinicians booked the room before the video visits.	The clinicians each used their own room, with their computer equipped with camera and headset.

**Table 2:** Number of observations and interviews conducted at Clinic A and Clinic B with clinicians, patient, relatives, or both patient and relatives.

Method	Total, n	Clinic A, n					Clinic B, n		
		Total	Clinician	Patient	Relative	Both	Total	Clinician	Patient
Observation	13	9	6	5	3	1	4	2	4
Interview	14	10	6	—^a^	—	—	4	2	—

^a^Dashes indicate patients or relatives were not interviewed.

## Results

### Overview

When clinics introduced video visits, we found that a new work task emerged while preparing for these visits, because the clinicians selected patients for video visits instead of offering them to everyone. The clinicians said in the interviews that they used different arguments for including or excluding patients for video visits. In addition, implicit reasons for inclusion or exclusion were identified in the observations. These reasons were based on the clinicians’ thoughts and assumptions regarding a patient’s condition and needs and on the content the clinician had planned or expected for the meeting. These arguments and implicit reasons can be understood as selection criteria for helping clinicians in their decision about whether or not to offer video visits to a patient. The selection was usually conducted individually by the clinicians, but sometimes they discussed different criteria and specific patients in advance with colleagues. Such discussions were both formal (during treatment conferences) and informal (chatting during breaks).

We identified 20 selection criteria, summarized in Table 3, that were either expressed in interviews, identified during observations, or both. Often several criteria interacted with each other. We divided the identified criteria into 3 themes: practicalities, patient ability, and meeting content*.* Each criterion is described, without any preferred order, under its respective theme. Due to the small number of respondents, we have not been able to evaluate the degree to which each criterion is relevant. This may also be dependent on the individual patient, on the diagnosis, or on the type of care.

### Practicalities

The criteria regarding practicalities are about the essential conditions that need to be fulfilled to achieve video visits. They are also related to clinicians’ desire to provide a good service for their patients. For example, for patients who find it cumbersome traveling to the hospital, video visits can facilitate the start or continuation of a treatment program. The criteria regarding practicalities were used by the clinicians to make an assessment from 2 perspectives: conditions and facilitating the treatment.

From the conditions perspective (1), 3 criteria needed to be fulfilled:

- the patient is positive about video visits
- the patient has access to the necessary technology
- the patient has had previous face-to-face meetings at the clinic

All 3 criteria are fairly easy to assess, as there can only be 2 answers: yes or no (eg, the patient either has the required technology or not). In 1 case, the family did not have access to a camera (1b), but they were so positive about video visits (1a) that they borrowed a tablet from a relative (Clinic A, Int_5). Furthermore, 1 clinician expressed this by saying:

They must be positive and have the right conditions for using the technology.

Clinic B, Int_11

It is important to bear in mind that having access to the technology does not imply being able to handle it (see 4d below). Regarding the technology, the clinicians need to have access to the requirements and be updated on any future changes. The third criterion (1c) is seen as a condition for being able to establish a relationship with the patient (5a), as shown below.

From the perspective of facilitating the treatment (2), 5 criteria were used to include patients for video visits. These criteria were used for patients:

- living far away from the clinic
- with economic issues that made it difficult to fulfill the care
- with other illnesses or disabilities that could be affected by physical visits
- with family, work, or school-related issues that complicated physical visits
- with lack of time due to school, work, or medical status

These 5 criteria could be answered with either “yes” or “no” and were thus easy to assess. However, what was considered a long distance to the clinic was a subjective assessment made by the clinician. The most commonly expressed reason for why a patient (or relative) was offered a video visit was related to having a long distance to travel to the hospital (2a). The clinicians discussed how long a time it would take for the patient and relative to get to the clinic, or where the patient lived. An hour or more of traveling time 1 way for the patient was not uncommon. The distance was sometimes also connected to lack of time (2e), for example, patients not wanting to be away from school. A long distance to the hospital (2a) could also affect how positive the patient is to video visits (1a):

...for this family, with a long distance [to the hospital] and several other contacts with health care, the mother has been very positive about meeting in this way instead [through video visits].

Clinic A, Int_1

Some clinicians also mentioned stigma and how the patient might suffer from leaving school for obesity treatment (2d). Leaving a lesson for a video visit was described as being less stigmatizing than leaving school for half a day to travel to the clinic.

One patient at Clinic B was considered to “really benefit from the video visits,” because he had physical difficulties getting to the clinic and had a weak immune system (2c), thus making him:

...very susceptible to infection [...] I think it’s a good service for such a patient, to save some of his energy and force.

Clinic B, Int_13

Clinicians hence offered video visits if there were practical issues making it difficult for the patient to travel to the hospital but also because the clinician wanted to protect the patient from uncomfortable situations. These criteria can be seen as fairly easy to assess. The patient’s fulfillment of some criteria in this theme may change over time. For example, patients might change their minds about video visits, get the right equipment, or move.

### Patient Ability

The criteria regarding the patient’s ability were used by the clinicians to make an assessment from 3 perspectives: the patient’s well-being, mindset, and relationship with the clinician.

From the well-being perspective (3), the following criteria were used:

- the patient is stable in weight
- the patient is mentally stable
- the patient does not have multiple diagnoses

**Table 3:** A summary of the 20 criteria divided into 3 themes.

Theme, perspective, and code			Criterion	
**Practicalities**				
	**Conditions**			
		1a		The patient is positive about video visits
		1b		The patient has access to the necessary technology
		1c		The patient has had previous face-to-face meetings at the clinic
	**Facilitating treatment**			
		2a		For patients living far away from the clinic
		2b		For patients with economic issues making it difficult to fulfill the care
		2c		For patients with other illnesses or disabilities that could be affected by physical visits
		2d		For patients with family, work, or school-related issues that complicated physical visits
		2e		For patients with a lack of time due to school, work, or medical status
**Patient ability**				
	**Well-being**			
		3a		The patient is stable in weight
		3b		The patient is mentally well
		3c		The patient does not have multiple diagnoses
	**Mindset**			
		4a		The patient can take responsibility
		4b		The patient has an understanding of the disease
		4c		The patient can understand instructions about how to use the technology
		4d		The patient is able to handle the technology
	**Relationship**			
		5a		The clinician has an established relationship with the patient
**Meeting content**				
	**Enablers**			
		6a		An expressed need, from patient or relative, for more frequent contact or encouragement
		6b		An identified need, from clinicians, for more frequent contact, more encouragement or feedback to achieve a better treatment outcome
		6c		Video communication adds something in comparison to other communication media
		6d		Video communication does not include sensitive issues

Criteria 3a and 3c are easy to assess as there can only be 2 answers, “yes” or “no.” Criterion 3b is more difficult to determine because if not diagnosed, it requires a subjective assessment by the clinician. For example, 1 reason given for not offering video visits to a patient who is mentally unstable was the uncertainty about what to do if the patient began to cry. The clinician wanted to have the opportunity to comfort the patient if they cried by giving them a handkerchief, a glass of water, or a pat on the shoulder.

Patients who were considered less suited for video visits were those with multiple diagnoses (3c):

In those cases where I feel no, it might not be relevant [with video visits] sometimes it depends on multiple diagnoses, how it will work out with the communication and instructions, and so on.

Clinic A, Int_5

This example also addresses criterion 4c below.

From the mindset perspective (4), the following criteria were used:

- the patient can take responsibility
- the patient has an understanding of the disease
- the patient can understand instructions about how to use the technology
- the patient is able to handle the technology

These criteria might be difficult to assess as they are subjective and depend on how well the clinician knows the patient. The assessment is therefore, to a large extent, grounded in the established relationship between patient and clinician and in the effects and progress of the treatment program. If the patient is fairly new to the clinician, and if there are other criteria that make the patient eligible for video visits, the trust in the patient’s ability to take responsibility (4a) needs to be established through other means, for example, first impressions when talking to the patient (1c).

To be included for video visits, the patients needed to be able to take responsibility for choosing a suitable location for the video visit and having their weight taken in advance. In other words, new tasks were introduced for the patients. Weight is an important parameter for the clinician to assess the treatment of the patient. For patients at Clinic A, 1 condition for being offered video visits was that the school nurse could take the weight and send the figures to the clinic, where it could be written into the medical journal. In 1 of our observations, the clinician asked and explained to the patient:

Did you take your weight with the school nurse [...]? It’s a requirement for video visits, that you go there, but the nurse cannot chase you.

Clinic A, Obs_2

For this specific patient, the video visits in combination with physical meetings were important to maintain the treatment; see criterion 6b below. In another example, from Clinic B, 1 of the clinicians reasoned around the patients’ ability to take responsibility (4a) based on personality, and the patients’ understanding of the disease (4b):

The patient is one of those who you cannot only have the video visit with [...], because I don’t know how the treatment would turn out. However, there are of course some others [...] who have the opposite personality, who are super organized and super skilled in that way, if [a specific patient] had had a somewhat fairly reliable scale at home, [the patient] would not have had to come here to be weighed.

Clinic B, Int_12

Uncertainty related to criterion 4a about responsibility was expressed during a lunch break when clinicians at Clinic A discussed what to do if a patient suddenly turned off the video and left the video meeting due to being upset.

From the relationship perspective (5), 1 criterion was used:

- the clinician has an established relationship with the patient

This criterion is subjective and depends on how well the clinician knows the patient (1c). Knowing about the patient’s ability to take responsibility (4a) implies that the clinician must have a well-functioning collaboration and an established relationship (5a) with the patient:

Those who I have been thinking about to offer video visits, are those I know well and who have been here for quite some time. I do not think a video visit is just as useful for new visits, when you may have only met the patient a few times. I think you must have developed some kind of treatment alliance and relationship and have explained the growth curves. It’s a feeling I get, and those I’ve chosen, these I know and feel that we have a relationship. We have some kind of treatment alliance. I think that it’s difficult to build a treatment alliance via video visit, and the treatment alliance is the foundation of our treatment.

Clinic A, Int_1

Using video visits with new patients was seen as less suitable because the relationship between the patient and clinician had not yet been established. In all cases but 1, the clinician had met the patient (or relative) face-to-face before the video visit (1c). In the 1 case where the clinician had not met the patient, the patient had previously met with another clinician at the clinic. It was stated that the relationship between a clinician and patient is established (5a) during physical meetings. Maintenance of a well-established relationship, however, was not considered to need close physical proximity, and video visits were therefore considered an option only when the clinician and the patient had already established a relationship.

Criteria about the patient’s ability can be summarized as follows: the patient having a personality suited for video visits, he or she is able to take responsibility, and there is an established relationship between the clinician and patient. Most of these criteria may change over time, for example, criterion 5a, as the relationship between clinician and patient can develop over time. Thus, this indicates that the assessment of who is considered suitable for video visits (or not) is dynamic over time. 

### Meeting Content

Criteria about the content of the meeting or the treatment plan concern aspects that can affect the outcome of the meeting or the treatment in the longer run. They were used by the clinicians to make an assessment from 1 perspective: being an enabler for the treatment program.

From the enabler perspective (6), the following criteria were used:

- an expressed need, from patient or relative, for more frequent contact or encouragement
- an identified need, from clinicians, for more frequent contact, more encouragement or feedback to achieve a better treatment outcome
- video communication adds something in comparison to other communication media
- video communication does not include sensitive issues

These criteria could be seen as driving forces for using video visits or enablers of the meeting content. Criteria 6a and 6b can be identified by the patient, relative, or clinician, by needs that may grow over time, during conversations and as the relationship develops. In the following example, the clinician identified a need for more frequent contact (6b) and suggested video visits to the patient, who then expressed the same need (6a):

I suggested [video visits] pretty quickly and directly they were very positive about this and they were also quite clear that, yes, we would like to have a little more frequent contact and pep talk.

Clinic A, Int_5

The possibility of offering more frequent contact, for example, by dividing a 1-hour meeting into 4 15-min meetings, is only practically possible through long-distance communication.

In contrast to the first 2 criteria, criterion 6c may rather be something that is triggered by a need while planning for the meeting. In the following example from our data, a planned face-to-face meeting was considered to benefit from being rescheduled into a video visit (6c):

...one of the clinicians first thought of just calling the relative, but instead chose to offer a video visit when she realized that she could show the growth curve in orderto calm the relative, showing that the patient was doing fine.

Clinic A, Int_10

Another example is when a clinician expressed the difference between using video and a phone (6c):

When the patients are younger it feels important to engage the parents, and video visits provide the ability to involve more people in the conversation compared to phone conversations.

Clinic A, Int_5

A third example is when the patient and the relative lacked time to attend a meeting (2e), but the clinician still wanted to see them to grasp their interplay (6c; Clinic A, Int_6).

Sensitive issues (6d) may be needs triggered while planning for the meeting and more difficult to assess; perhaps not in terms of what may be sensitive or not, but whether a video visit is suitable for that specific issue or not. The presumption of the technical limitations of using video visits, therefore, generated selection criteria regarding what kind of consultation the clinician thought would be useful; for example, the fact that sensitivities could not be included in the treatment session using video visits. However, there seemed to be a difference in these presumptions between experienced and inexperienced clinicians. For the clinicians at Clinic B, who had conducted video visits several times during a period of 1 year, this did not seem to be an issue. One clinician said that when implementing video visits, they worried about whether the technology would work or not and, therefore, did not have as much focus on the patient. However, now while being used to the technology and trusting it to work, the clinician felt that they were able to catch the patients’ mood even if they were not located in the same place (Clinic B, Int_14). Being able to include sensitive issues in the video visit could interact with criterion 4a: the patient’s ability to take responsibility regarding their choice of environment. A schoolyard, for example, was not seen as a proper place for the patient when discussing certain issues.

Usually, the clinicians stated several different reasons for offering video visits, reasons that could be connected to several of the criteria: “The mother had just had a baby” (2d), “they live far away” (2a), “and we need fairly close contact. Every second or third month doesn’t work here” (6b; Clinic A, Int_2). In some cases, video visits may be necessary to meet criteria 6a, 6b, and 6c; that is, if more frequent contact is needed, then it may only be possible through video visits. Whether or not to suggest video visits to a patient also depended on the content of the meeting. Some of the criteria may be identified while planning for the meeting.

### Summary of Findings

Table 3 summarizes the criteria identified and described above, divided into 3 themes. There is no specific order or strength in the criteria, that is, no one is more important than the others. However, in specific situations, when the clinician is assessing the criteria, some may be seen as more valid or more important than others.

## Discussion

### Principal Findings

The clinicians did not consider all patients suitable for video visits, which added the new work task of selecting patients [5]. When doing so, the clinicians relied on their understanding and knowledge about the patient and his or her progress during treatment. While assessing whether a patient was suitable for video visits, the clinicians developed and used different criteria. We identified 20 criteria, which we themed under practicalities, patient ability, and meeting content. The criteria can be seen as requirements involving the patients’ external and internal circumstances. External circumstances, that is, most criteria under practicalities, relate to the patients’ surroundings and environment and appear easy to assess (eg, access to technical equipment and distance to the clinic). Internal circumstances, that is, most criteria under patient ability, relate to the patient’s needs and abilities (eg, the patient has to be mentally well and able to manage the technology). External and internal circumstances can change over time, with the effect that the assessment may change and result in a different decision.

### Assessment of the Criteria

Video visits, which involve geographical distance between clinician and patient, provide good opportunities for qualitative care [1-3,5,10-12]. We found that the criterion about the patient’s distance from the clinic was a strong incentive for the patient to use video visits instead of face-to-face meetings, thereby becoming the most common inclusion criterion used by the clinicians. We also found other criteria concerning different practical conditions, the patient’s abilities, and the meeting content that, to a greater or lesser extent, influenced the possibility to conduct video visits and their suitability. Some criteria could easily be assessed by a yes or no, for example, whether the patient had the required technology or not. Other criteria required a more qualitative and subjective assessment of the patient’s needs and abilities, for example, a need for more frequent contact. Some assessments were based on interpretations, for example, whether the patient could understand instructions. From the clinician’s perspective, knowing about the patient’s needs and abilities required an established relationship with the patient.

Usually, the clinicians stated several reasons for offering video visits. Several criteria thus interacted in the assessment and during the selection process. Some criteria could be reinforced by others, and some were considered more important. For example, if the patient was not stable in weight (a strong indication for not offering video visits), lived far away from the clinic, and the clinician had identified a need for more frequent contact (2 strong indications for offering video visits), the patient might still be selected for video visits. Similarly, some criteria could exclude video visits even though other important criteria were fulfilled. For example, if the patient did not have the technical equipment required, it would not be possible to conduct video visits even if the patient wanted to and was mentally well.

The assessment was made by the clinicians, often on their own and sometimes in discussion with a colleague based on their knowledge about and trust in the patient. Both knowledge and trust are built through the relationship between clinician and patient. Trust is also related to the patient’s abilities—something the clinician learns as the relationship develops. Research from other studies indicates that the perception of video visits may differ between the clinician and patient [15]—something that may coincide with the relationship.

### Consequences of the Selection Process

The clinicians at both clinics chose to take responsibility for selecting patients for video visits. Our interpretation is that they did this simply because they wanted to achieve the best possible outcome from the treatment [5]. What would happen if the patient could make the choice of having video visits by themselves—not spontaneous meetings [5] but meetings as part of the treatment program? It seemed that the clinicians did not see this as an option, mainly because it could have a negative effect on treatment. However, it may be difficult for clinicians to fully understand “the patients’ lives” [5] and thereby make a proper assessment. Selecting patients for video visits implies that some patients will be excluded and thus not given the same opportunity due to differing circumstances [4]. The question is how the patient’s needs and desires can be taken into account without having a negative effect on the treatment. The complexity of the meetings within the treatment program makes it reasonable to think that clinicians should have a large say in deciding whether a meeting should take place face-to-face or as a video visit [5].

Some of the criteria used for deciding whether a patient is suitable for a video visit or not have to do with distance or whether it is cumbersome for the patient to travel in any other way. How do the clinicians balance between such aspects, which can be highly relevant for the patient, and other criteria that, for example, could have an effect on the content of the meeting? How do they balance between what the patient wants and what they think the patient can manage and still achieve good results in the treatment program? The clinicians need time to find this level of balance [5] to understand the transformation of roles and responsibilities [12] and how these issues affect the content of the treatment program. The selection criteria developed and used by the clinicians, together with influence from or even in collaboration with the patient, can be a tool for finding this balance, something that we expand upon below.

#### Practicalities

The criteria within the theme practicalities include both conditions that are necessary for conducting video visits and aspects that can be seen as enablers for the patient to fulfill or undergo the treatment at all. To a large extent, the enablers may influence the clinicians in their decision, especially if the patient lives far away from the clinic, if they cannot get away from work or school, or if there are other family-related issues that may complicate face-to-face meetings. If the incentive for the patient is strong regarding these criteria, then the clinician may need to overlook other criteria related to the patient’s abilities, just to be able to proceed with the treatment.

#### Patient Ability

The criteria within the theme patient ability include aspects regarding the patient’s well-being and mindset and the relationship between patient and clinician. Several of these criteria are closely related to trust and judgment. When video visits were introduced, responsibilities were transferred from the clinician to the patient, for example, taking their weight. The clinicians were dependent on knowing the patient’s weight to judge how well the treatment was proceeding. Thus, this new responsibility for the patient influenced the clinician’s ability to follow the patient’s progress in the treatment program, which in turn affected the clinician’s assessment of the selection criteria. The new responsibility for the patient implies that the clinician has to trust the patient’s ability to fulfill this responsibility.

Using video visits, the patient’s nonclinical place and space is added to the meeting [10-12]. It was the patient’s responsibility to choose his or her location for the video visits, a location often unknown to the clinicians beforehand. The patient’s location, including its surroundings, sometimes resulted in disturbances and limitations that in turn could affect the meeting [12]. Sometimes the clinicians had to adjust the content of a meeting to allow for disturbances and limitations situated in the patient’s location. For example, if a planned session includes sensitive issues to be discussed, these can instead be brought up in a later meeting that is conducted either face-to-face or as a video visit with the condition that the patient chooses a more suitable location. The clinicians based their selection on their knowledge of the patient’s ability to choose a location with as few disturbances or limitations as possible. Hence, the patient’s ability to take responsibility for their place and space influenced the content of the meeting, which in turn affected the assessment of the selection criteria.

Assessing the patient’s ability to take responsibility for selecting a suitable location or taking their weight might not be easy. If the patient has an understanding of the disease, his or her ability to take on the responsibilities that come with the new role may increase. Issues related to responsibility can, however, be difficult for the clinician to communicate and for the patient to understand. Still, such aspects need to be considered by the clinician when selecting a patient for video visits. Knowing the patient is 1 condition for understanding their ability to take responsibility; an understanding that is subjective and related to several criteria. An established relationship with the patient is therefore necessary and might also be a condition for the consultation to work better [5]. Face-to-face meetings with the patient were considered necessary to establish a relationship. This might grow over time, which means that the clinician’s assessment of the patient’s suitability for video visits may also change over time.

#### Meeting Content

The criteria within the theme meeting content include needs related to more frequent contact, the possibility to add content to the meeting that cannot be added using other communication media, and sensitive issues. These criteria are more complex and are affected by issues of a more subjective character, such as trust. Either the patient or clinician could require more frequent contact or encouragement; for example, a 1-hour meeting could be divided into 4 15-min meetings. In addition, being in different locations could add new content to the meeting, for example, providing the clinician with a view into the patient’s home and thereby open up the patient’s private sphere [12]. This gives the clinician an opportunity to meet the patient in his or her own context, adding new details to the treatment program and to the clinician-patient relationship. Video visits could thus generate content in the meeting that would not be practically possible in face-to-face meetings. If sensitive issues were to be discussed, this could affect the clinician either in the selection process, for example, scheduling that specific meeting as a face-to-face meeting, or in the planning process, for example, bringing up the sensitive issues in the next meeting.

### Using the Criteria as a Tool in the Selection Process

Introducing video visits changes ordinary work practices [5], and this in turn can affect the conduct of the meeting with a risk of introducing disturbances and limitations [5,12]. Greater requirements on the patient’s abilities could reduce this risk. For example, if the patient can handle the technology, then the risk of the clinician becoming first technical support is reduced [5,12]. Similarly, if the patient can take responsibility, the risk of ending up in a meeting with a large number of unaccounted-for disturbances is reduced [5,12]. Hence, the criteria used in the selection process can reduce the number and severity of disturbances and limitations in the meeting, but the criteria can also be used to communicate the requirements they represent to the patient.

The criteria developed for and used in the selection process can, to some extent, be used to guide the clinician in how to think during this selection process. However, the reality is never easy, which means the selection process is intertwined with pros and cons when deciding whether the patient is suitable for video visits or not. Our interpretation is that the selection process is based to a large extent on the clinician’s thoughts, knowledge, and assumptions regarding the patient and his or her abilities [5]. We cannot give a clear-cut answer to what would happen if the patient could decide on video visits or at least be an active part in the selection process. Greater patient involvement in the selection process may strengthen the patient’s sense of responsibility and perhaps also reduce the difference in perception of the video visits [15]. Several of the criteria used in the selection process involve the patient’s opinion, but it is still the clinician who makes the assessment and decides. Perhaps the patient’s ability to take responsibility could be strengthened if the patient was more involved in the selection process. This could increase the patient’s understanding of the new responsibilities. A first bonding, during which the clinician and patient establish a good relationship, appears to be necessary, however. Methods for involving the patient in the assessment of the criteria may also need to be developed.

### Conclusions

We conclude that not all patients, adults, or children with obesity undergoing treatment programs should be offered video visits. The patient’s new responsibilities of choosing a suitable place and taking their own weight could influence the content of the meeting and the progress of the treatment program. The selection criteria developed and used by the clinicians could be used as a tool for finding a balance between what the patient wants and what the clinician thinks the patient can manage and achieving good results in the treatment program. The criteria could also reduce the number and severity of disturbances and limitations in the meeting and be used to communicate the requirements they represent to the patient. Some of the criteria are based on facts; for example, if the patient does not have access to the required technology, then a video visit is not an option. Other criteria are subjective; that is, they are more formal and used rather as a checklist to avoid individual interpretations among the clinicians. A method for how and when to involve the patient in the selection process is recommended since it may strengthen the patient’s sense of responsibility and relationship with the clinician. 

### Implications for Further Research

Further research is required to understand the full effects of selecting patients for video visits. Our study provides 1 piece of the puzzle and can guide other researchers in studying the selection task. We have focused on a small path of patient flows, a chronic disease for which the patients undergo a treatment program with several consecutive meetings. There are no data that need to be monitored daily; only a little data collected by the patient are used to follow the progress of the treatment. The selection criteria are likely to differ in other patient flows and in other types of care programs. Due to the low number of respondents in the study, we can neither prioritize the criteria nor generalize the results. Instead, our results can be seen as something upon which further research can be built. We have not measured the specific benefits of video visits compared with face-to-face visits regarding the progress of the treatment, and we know that the values may differ between diagnosis areas. A health economics study of the benefits, for different stakeholders, would be most interesting.
